# Organizational democracy and meaningful work: The mediating role of employees corporate social responsibility perceptions

**DOI:** 10.3389/fpsyg.2022.946656

**Published:** 2022-11-22

**Authors:** Mari Svendsen, Thomas Faurholt Jønsson

**Affiliations:** ^1^Department of Leadership and Organization, Kristiania University College, Oslo, Norway; ^2^Department of Psychology and Behavioural Sciences, School of Business and Social Sciences, Aarhus University, Aarhus, Denmark

**Keywords:** organizational democracy, participation in decision making, corporate social responsibility, meaning at work, meaningfulness at work, alienation

## Abstract

Meaningful work is an important field of research, relating to both organizational outcomes and employee welfare. Organizational democracy has been theoretically proposed as an important antecedent to meaningful work. Nevertheless, this relationship is yet to be empirically explored. Thus, the objective of the current research is to explore the relationship between organizational democracy and meaningful work. We used structural equation modeling with self-reported, cross-sectional data from different nations and industries to test a mediation model in which corporate social responsibility (CSR) perceptions mediate the positive relationship between organizational democracy and meaningful work. Our findings confirmed that CSR perceptions partially mediate in the relationship between organizational democracy and meaningful work. Thus, based on our findings we can conclude that organizational democracy can play a direct role in increasing the experience of meaningful work, but also an indirect role trough the employees experience of CSR. Our findings have theoretical implications by adding to the classical theoretical literature that connect organizational democracy and meaningful work, and by disentangling the role of CSR perceptions in this relationship. Moreover, our findings have practical implications as our results give important knowledge to managers and organizational stakeholders that wish to increase the experience of meaningful work in organizations.

## Introduction

*“Striving for meaningful work is not just about obtaining certain outcomes, but being able to experience meaningfulness in one’s work activities is “an important humanistic endeavor in and of itself”* (Lepisto and Pratt, 2017, p. 100), *and “part of what makes life good for human beings”* (Yeoman, 2014).

As the above quotations describe, the importance of meaningful work has historically been recognized as crucial for human thriving and growth, whereas a lack of meaning and alienation from work has been described as toxic for human welfare (Marx, 1959; Shantz et al., 2014). For example, the experience of meaningful work is positively related to work motivation, work engagement, life meaning, and general health, and negatively related to negative affect, mortality, and withdrawal intentions (Allan et al., 2019). Meaningful work can be defined as the global judgment that one’s work accomplishes significant, valuable, or worthwhile goals that are congruent work with one’s existential values (Allan et al., 2019, p. 502). Due to the importance of meaningful work for individual welfare and organizational effectiveness, the question of how to create meaningful workplaces is a pertinent one. Accordingly, the main motivation of this study is to obtain a better theoretical and practical understanding of factors that may increase employees experience of meaningful work. Marx (1959) described a rigid division of labor and the ensuing lack of control in the workplace, along with the inability to develop and use one’s skills and knowledge, as an important source of alienation and meaninglessness in the workplace. In extension of this argument, democratization in the workplace has been proposed as an important factor that may reduce the alienating consequences of nonautonomous work and some authors have theoretically argued that workplace democracy is a necessary requirement for meaningful work (Yeoman, 2014; Frega et al., 2019). Organizational democracy refers to ongoing, broad-based, and institutionalized employee participation that is not *ad hoc* or occasional in nature (Weber et al., 2020, p. 1009). Although organizational democracy has, both historically and theoretically, long been suggested as an important antecedent of meaningful work, there has not, to the best of our knowledge, been any *empirical investigation* of this relationship. There are some indirect empirical evidence suggesting that factors closely related to organizational democracy, such as autonomy and power sharing may be positively related to meaningful work (Jin and Drozdenko, 2010; Martela et al. 2021). However, the direct relationship between organizational democracy and meaningful work has yet to be investigated and constitutes a profound research gap. This research gap is important to cover, in order to disentangle the potential that organizational democracy may have for the experience of meaning in the workplace. Moreover, as highlighted by Rosso et al. (2010), there is also a research gap, pertaining to the exploration of how different sources of meaningful work stimulate the experience of meaningfulness in the workplace simultaneously. Therefore, it is imperative to explore the mediating factors that may affect this relationship. Accordingly, the objective and the novelty of this article is to explore the relationship between organizational democracy and meaningful work and explore a mediating factor in this relationship.

In order to accomplish this goal, we develop a mediation model in which we propose that organizational democracy will be positively related to meaningfulness in the workplace. Moreover, we propose that the employees’ CSR perception, defined as *the degree to which employees perceive a company supports the activities related to a social cause* (Lee et al., 2013), will mediate the relationship between organizational democracy and meaningful work. CSR perception may be especially relevant when investigating the relationship between organizational democracy and meaningful work. Meaningful work is strongly connected to the individual’s values and the feeling that one’s work is in accordance with these values. Moreover, recent conceptualizations of meaningful work highlight greater good motivation as a central part of the meaningful work concept (Lee et al., 2013). Previous research has established the relationship between CSR perceptions and meaningful work (Raub and Blunschi, 2014), but the relationship with organizational democracy is yet to be explored. We designed the study as a cross sectional survey with temporal separation (2 weeks) of independent and dependent variables.

The exploration of the mediation model outlined in the present study will make three specific, novel and significant contributions to our understanding of both organizational democracy and meaningful work. First, we extend previous research on meaningful work by investigating how organizational democracy is directly related to perceived meaningful work. By doing so, we meet the call for a more thorough understanding of how the organizational context contributes to the perception of meaningful work (Lysova et al., 2019) and gain insights into a relationship that has historically been proposed as important but lacking direct empirical support. Second, we contribute to the understanding of organizational democracy by investigating how organizational democracy may lead to certain individual-level outcomes, responding to the call for research expressed by Weber et al. (2020). In their meta - analysis they find that organizational democracy is related to individual – level outcomes such as job satisfaction, work motivation and value-based commitment. Therefore, they conclude that organizational democracy may be especially suited to produce individual – level outcomes and call for research that consider other relevant individual – level outcomes. Our study will respond to this call for research and contribute to the understanding of the outcomes of having a organizational democratic work context. Third, our study contributes to the understanding of the mechanisms of which organizational democracy exerts its individual level influence in general. Weber et al. (2020) propose that organizational democracy may have a socializing effect on their employees. In accordance with that, we propose that the employees experience of CSR will be stimulated through a democratic work context. However, the relationship between organizational democracy and CSR perceptions is yet to be explored empirically. Thus, our study will also play a role in our conceptual understanding and development of organizational democracy.

The structure of the next sections of the paper is as follows. First, we will give a brief review of the empirical and theoretical literature that connects organizational democracy and meaningful work, and the relationship between organizational democracy and CSR and the relationship between CSR and meaningful work. Second, we will describe the methodology in detail. Third, the main findings of the study are discussed. Lastly, the implications for practice further research and conclusions will be presented.

## Theory and hypothesis

### Meaningful work

The following section will present literature on meaningful work. First, we define meaningfulness in a way that also corresponds with our measurement of the concept. Second, we review literature on the concept that is relevant in to understand the relationship between meaningful work and organizational democracy and CSR. Meaningfulness at work has been defined in various ways, with different streams of literature focusing on varying aspects of the concept (Martela and Pessi, 2018). A recent review by Martela and Pessi (2018) argued that meaningful work can be distinguished into three different categories: *significance, broader purpose,* and *self-actualization*. *Significance* refers to the amount of intrinsic value people assign to or are able to find from their work. *Broader purpose* refers to the idea that the work must contribute to some “greater good,” something beyond individual’s own benefits. Lastly, *self-actualization* refers to self-connectedness, authenticity, and how much we are able to realize and express ourselves through our work (Martela and Pessi, 2018, p. 3). In the literature on meaningful work there is a difference between *meaning* and *meaningfulness* (Rosso et al., 2010). The perception of meaning refers to employee’s cognitive evaluation of their environment, and can therefore be positive, neutral, or negative. The concept of meaningfulness, on the other hand, is inherently positive and refers to the experience of work as personally significant and worthwhile (Pratt and Ashforth, 2003). Another important distinction in the literature on meaningful work is that between *meaningfulness in the job*, which refers to the experience of meaningfulness in executing the work roles and *meaningfulness at the job*, which refers to the experience of being part of a social category. Accordingly, meaningfulness is a complex and multifaceted construct and is not a fixed property of a job or organization. However, Pratt and Ashforth (2003) argued that there are some socially constructed archetypes within a society or social group that the individual belongs to. Moreover, they argued that the process of which the work is experienced as meaningful shares similarities across organizations and cultures. A main argument from Ashford and Pratt (2003) is that because meaningfulness at work is partially socially constructed, and because the process of experiencing meaning share similarities between organizations and societies, organizations can play a key role in influencing whether and how organizational members view their work as meaningful. Ashford and Pratt (2003) argued that there are several different ways in which organizations can influence the experience of meaningfulness in the organizations, such as through leadership processes, the creation of psychological safety, and, importantly, through employee involvement practices.

### Organizational democracy and meaningful work

A concept strongly related to employee involvement practices is organizational democracy. In this section we first define the concept of organizational democracy in line with the measurement of the construct. Second, we will review both classical theoretical literature and recent empirical work to argue for the relationship between organizational democracy and meaningful work. Organizational democracy describes organizations wherein participative decision making is mandatory and realized either directly or indirectly (Pircher Verdorfer and Weber, 2016). Importantly, decision making in democratic organizations is not limited to the short-term decisions on an operational level, but also entails influence on long-term decisions at the strategic level (Pircher Verdorfer and Weber, 2016, p.61). According to the recent meta-analysis by Weber et al. (2020), indicators of organizational democracy can be conceptualized and measured in three ways. Firstly, through structurally anchored employee participation in organizational decisions. This indicator of organizational democracy focuses on the organizational level and is concerned with *democratic enterprise structures* such as self-governed employee-owned enterprises. The second way is through employee participation in collective ownership. This indicator focuses on *property* and is concerned with the degree to which employees own a substantial part of the shares in the enterprise. The third way is individually perceived employee participation in organizational decision making. The final indicator of organizational democracy focuses on the individual level in the organization and the extent to which the *employees perceive* that they have direct or indirect participation in strategic or tactical decision making.

Although, to the best of our knowledge, no empirical studies have investigated the relationship between organizational democracy and meaningful work, the relationship has been given theoretical attention. For example, Arneson (1987) argued that the experience of meaningful work is *“attached to a job that gives the worker considerable freedom to decide how the work is to be done and a democratic say over the character of the work process and the policies pursued by the employing enterprise”* (p. 522). In line with this, Yeoman (2014) argued that organizational democracy may be viewed as the institutional conditions that are required to achieve autonomy, which in turn are found to be imperative to the experience of meaningful work.

We argue that organizational democracy, as perceived by the employees, may directly strengthen the experience of meaning in two specific ways. First, we follow the classical work on alienation by Seeman (1959), who outline meaningfulness as one of the elements of alienation. In his analysis, meaningfulness is based on the perception of the world as comprehendible enough to form realistic expectations about how to control it. Since organizational democracy provides some control over the organization to its organizational members, participating employees may likely obtain a deeper and clearer understanding of how the organization is and why organizational decisions became as they did (Heller et al., 1998). This deeper, processual understanding of the organization may provide a meaningfulness organizational context to the employee.

Second, the experience of organizational democracy may stimulate the basic needs that are proposed by self-determination theory (the need for autonomy, the need for relatedness, and the need for competence; Deci et al., 2017), which are found to be associated with the experience of meaningful work (Martela et al. 2021). Martela et al. (2021) argued that the experience of ownership and autonomy at work makes work feel personally meaningful for the employee. Moreover, organizational democracy may stimulate the need for competence through mastery experiences and information sharing generated by employee involvement in strategic and tactical decisions (Weber et al., 2020). Lastly, the need for relatedness may be stimulated through cooperative decisions and collective identities stemming from organizational democracy. In a longitudinal investigation Martela et al. (2021) confirmed the relationship between the self-determination framework and the experience of meaningfulness at work, specifically underlining the strong positive relationship between autonomy and meaningful work (Martela et al. 2021). Thus, we posit the following hypothesis.

*Hypothesis 1:* Organizational Democracy, as perceived by the employees, are positively related to the experience of meaningful work.

### The effect of organizational democracy on employees perception of CSR

In this section we will briefly present the concept of CSR. We focus on the employee’s perception of CSR practices in the organization. CSR activities have been defined in different ways in the literature. Some researchers have focused on the organization’s behavior toward its stakeholders, whereas others have focused on the organization’s voluntary activities relating to social, political, environmental, and ethical actions (Malik, 2015). We adopt the societal perspective and follow Lee et al.’s (2013) definition of CSR as *“the company’s activities and status related to its perceived societal or stakeholder obligations”* (p. 1717). In line with this definition, the organization focuses on both the good of society and the welfare of the organization and its members. We follow Lee et al.’s (2013) definition and understand *CSR perceptions* as *“the degree to which employees perceive a company supports the activities related to a social cause” (p. 1717).* Moreover, we understand and treat employee CSR perceptions as a second order construct and choose to focus on the two sub-dimensions; philanthropic and ethical CSR activities (Lee et al., 2013). Although environmental perspectives are a vital part of the CSR understanding (c.f Lăzăroiu et al., 2020). We have explicitly chosen not to focus on this, as previous classical literature relating to organizational meaning keeps more focus on philanthropy and ethics.

Organizational Democracy and CSR are two distinct but interrelated concepts. Banerjee (2014) argued that democratic organizations are necessary for proper CSR in organizations. In line with this, Hazarika (2013) argued that *“in order to make the workforce more participatory, the firm has to uphold the principles of democracy in workplace practices and this will be a sheer move towards democratic social responsibility” (p. 12).* We argue that organizational democracy, which is ingrained in values signaling equality and participation, may induce positive organizational CSR perceptions by making the employees consider these values while analyzing the CSR practices of the organization. To the best of our knowledge, no empirical studies have investigated the relationship between organizational democracy and CSR perception. However, a quantitative study by Jin and Drozdenko (2009) found that CSR in general is strengthened by power sharing, open information sharing, and democratic ideology in the organizations. Accordingly, we propose the following hypothesis:

*Hypothesis 2:* Organizational democracy, as perceived by the employees, is positively related to the employees’ CSR perceptions.

### The effect of CSR perceptions on the employee’s experience of meaningful work

We further argue that CSR perceptions relate positively to the experience of meaningfulness at work. Rosso et al. (2010) argued that employees experience meaningfulness when they work for organizations that are engaging in socially responsible activities and see themselves contributing to a greater cause. Perceiving that the organization has high CSR may indirectly give employees the feeling that they are contributing to the economic, ecological, or social environment around them. Moreover, based on social identity theory, we know that individuals seek to identify with groups that contribute positively to their sense of self-worth (Tajfel, 1979). Having a strong perception of the organizations CSR activities will contribute to the experience that the organization has values that are useful and worth identifying with, and through that the employees experience a sense of belonging and meaningful co-existence. Recent studies support the positive relationship between CSR and the experience of meaningfulness at work (Chaudhary, 2020; Youn and Kim, 2022). Accordingly, we posit the following hypothesis;

*Hypothesis 3:* Employee’s perception of CSR is positively related to the experience of meaningful work.

### The mediating role of CSR perceptions

Organizational democracy may influence the experience of meaningful work directly through the creation of especially greater autonomy and by reducing the potentially alienating consequences of work. Nevertheless, favorable CSR perception may be conceived as an innovative mechanism that transmits the influence of on organizational democracy on meaningfulness at work. Accordingly, organizational democracy can be anticipated to affect meaningfulness both directly as well as indirectly *via* CSR perceptions. Thus, we propose the following hypothesis:

*Hypothesis 4:* Employees’ perceptions of CSR partially mediate the relationship between organizational democracy and the experience of meaningful work.

**Figure 1:**
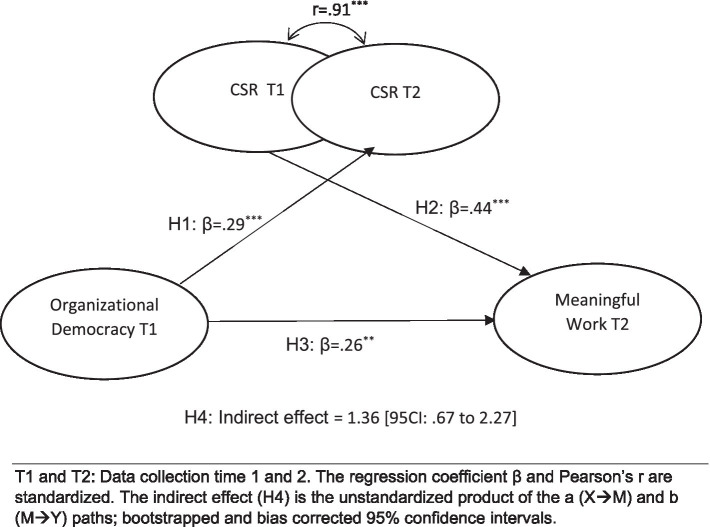
Hypothesized Model and results T1 and T2: Data collection time 1 and 2. The regression coefficient *β* and Pearson’s r are standardized. The indirect effect (H4) is the unstandardized product of the a (X → M) and b (M → Y) paths; bootstrapped and bias corrected 95% confidence intervals.

The proposed hypotheses are shown in an overall model (Figure 1).

## Materials and methods

### Sample and procedure

The survey was obtained *via* Prolific, which is a company with extensive experience in providing data to research institutions. Previous research has shown that similar data collection methods provide better external and internal validity than traditional data collection methods (Berinsky et al., 2012). In our sample, every participant came from different organizations, so our sample did not violate the independence assumption that may result in spuriousness due to data clustering (Raudenbush and Bryk, 2002). Mean age was 31.1 (SD = 10.8). Six respondents had Elementary School level, 62 graduated from High School. 26 had finished undergraduate studies, 62 had a Bachelor’s Degree and 54 had a Master or higher level of education.

We designed the study as a cross sectional survey with temporal separation (2 weeks) of independent and dependent variables (Podsakoff et al., 2012). The reason was to deal with potential inflation of relationship estimates due to cross sectional data collection at the same time, we collected the data two times with a few weeks between the two data collections. We applied the data from T1 to measure the independent variable and the data from T2 to measure the dependent variable. We used the mediator measures from both times with the purpose to deal with common method bias (see Figure 1). Specifically, we analyze the hypothesized relationships between variables from different data collection times, i.e., relationships between the mediator and both the independent variable and the dependent variable. We tested a structural equation model (SEM), in which the independent variable (measured at T1) was related with the mediator from T2, and the mediator from T1 was related with the dependent variable (from T2). In this way, the variables of any relationship were measured at different time points. Hence, we avoid method inflation of the regression estimates due to measurement at the same time point (i.e., mood effects and other effects of situational mental states). Since we applied measurements the mediator from two time points, we restricted the factor loadings to be the same for both measurements of the mediator variable (“metric invariance”). Thereby, we ensured that the two measures of the mediator were fully comparable, and that different ways of measuring the mediator at T1 and T2, respectively, contaminated the regression coefficients. Since the same items from the CRS variables measured at T1 and T2 would potentially hold a similar item specific measurement error, we allowed the same items to correlate in the model.

We tested measurement validity using Confirmatory Factor Analyses (CFA). If a Modification Index (MI) analysis showed a substantial improvement of model fit (MI > 10.00) by allowing items within a factor to correlate or a theoretical meaningful cross-loading of an item on to two sub-factors, we allowed this in a subsequent model. To assess reliabilities of the scales, we applied the composite reliability, which is comparable to but more precise than Cronbach’s Alpha because the former does not assume that all loadings in a factor are of equal size (Raykov, 1997). Finally, we included control variables to test for possible confounding variables. We included age, education (binary coded dummy variables), and gender to control for the possibility that age, educational level or gender *per se* would provide the employee with more influence in an organization, gives a stronger perception of CSR and make the job feel more meaningful. We applied the statistical software Mplus v. 8.2 (Muthén and Muthén, 2017) for structural equation modeling (SEM) using maximum likelihood (ML) estimation. ML estimation assumes multivariate normal distribution though it is even consistent in violations of normality. Therefore, it is useful for the present study that have a suitable large sample size and assumed normality of the variables. We used the most applied fit indices and cutoff criteria, namely RMSEA (<0.08) and CFI (>0.95; Hu and Bentler, 1999; Schermelleh-Engel et al., 2003).

### Measures

All the applied scales in this study had been previously published and validated. All continuous measures were assessed on a five-point Likert scale, with responses ranging from 1 (strongly disagree) to 5 (strongly agree).

#### Organizational democracy

We measured Organizational Democracy using the 24 item Short Version of the Perceived Structure of Organizational Democracy Questionnaire (Weber and Unterrainer, 2012; POPD-S). The scale consists of three subdimensions measuring influence on issues at the operational level (employees’ daily work or working time), the tactical level (e.g., employment policies or innovation of work processes), and the strategic level (financial decisions, strategic planning, election of leaders and new shareholders). A CFA confirmed the three dimensional scale structure (*χ*^2^(54) = 122.98, RMSEA = 0.076, CFI = 0.965). We also estimated the Composite Reliability (CR), to be.96, which is well beyond the.70 rule of thumb for acceptable reliability.

#### Corporate social responsibility perceptions

CSR perceptions included Philantropic CSR Activities and Ethical CSR Activities and we measured this construct using 11 items from the scale by Lee et al. (2013). Moreover, we found that two items (no. 7 and 8) loaded on the first subfactor rather than the second as originally suggested by Lee et al. (2013). Therefore we moved these two items to the former subscale.

An example item is “Our company has a strong sense of corporate social responsibility.” A series of CFAs showed that residual variance between item 1 and item 2 as well as item6 and item 7 correlated, which we accepted. The process ended with acceptable fit indices (*χ*^2^(41) = 95.67, RMSEA = 0.080, CFI = 0.954). Reliability was also good (CR = 0.92). At T2, we obtained a satisfactory fit for the same model and the same item error correlations (*χ*^2^(41) = 72.97, RMSEA = 0.061, CFI = 0.968), and CR = 0.91. The scales correlated very highly (*r* = 0.91), and this result supports that there is a high degree of test–retest reliability of the scale.

#### Meaningful work

Meaningful work was measured using a 10-item scale developed by Steger et al. (2012). The scale consists of three subscales measuring positive meaning, meaning making trough work and greater good motivation. An example item is “I understand how my work contributes to my life’s meaning. Modification Index (MI) analyses revealed that the item “I view my work as contributing to my personal growth” cross-loaded on the Positive Meaning sub-factor. A SEM analysis showed acceptable fit indices of a model with the cross-loading item (*χ*^2^(31) = 74.37, RMSEA = 0.082, CFI = 0.966). In addition, the analysis showed that the reliability was good (CR = 0.93).

## Results

Table 1 reports the descriptive statistics and correlations among the variables and Average Variance Extracted. The two latter estimates were based on a Measurement Model, which demonstrated satisfactory fit: *χ*^2^(883) = 1413.93, RMSEA = 0.053, CFI = 0.91. We computed the AVEs for the hierarchical constructs as the means of the completely standardized squared loadings of the first order dimensions (following MacKenzie et al., 2011). The results show that the AVEs are higher than all intercorrelations (Fornell and Larcker, 1981), with the exception from CSR perceptions at the two time points. The latter should not differ because it is the same construct. All in all, the results support the expected discriminant validity of the different variables (Table 2).

**Table 1:** Descriptive statistics: mean, SD, Pearson’s correlations, reliabilities.

	Mean	SD	1	2	3	4	AVE
1. Organizational Democracy	2.77	0.89	(0.96)				0.65
2. CSR Perceptions T1	2.99	0.91	0.35^***^	(0.92)			0.70
3. CSR Perceptions T2	2.97	0.87	0.29^***^	0.91^***^	(0.91)		0.78
4. Meaningful work	3.46	0.78	0.41^***^	0.53^***^	0.60^***^	(0.93)	0.73

All variables’ answer scales range from 1 to 5. Means and SDs calculated from scale means of manifest item values in SPSS v. 28. Pearson’s Correlations between latent variables and Average Variance Extracted (AVE) was computed on basis of the Measurement Model. Composite Reliability across the diagonals in parentheses.

*** *p* < 0.001.

**Table 2:** Items, subscales and standardized loadings.

Organizational democracy (Weber and Unterrainer, 2012)		
How much influence do you experience on	Standardized loadings	
*Subscale: Operational area*		
1.How the daily work is organized	0.94	
2.How the daily work tasks are organized	0.90	
3.How working time is organized and scheduled	0.70	
*Subscale: Tactical area*		
4.The employment policies of the organization	0.76	
5.Purchasing of new resources and equipment (e.g., machinery and tools, information technology/media)	0.63	
6.How health and safety are managed	0.72	
7.Process innovations (e.g., extensive improvements of technology, work organization)	0.73	
*Subscale: Strategic area*		
8.Appointment of a new head of department/division or of your direct superior,	0.84	
9.The financial decision-making by the organization (e.g., concerning the budget of the firm, major capital investments or applying for credit)	0.85	
10.Plans and strategies for the development of the organization (e.g., corporate constitution and governance, mission statement, restructuring of the company)	0.86	
11.Election of the chief executive or members of the executive board or supervisory board	0.89	
12.Admission of new shareholders, stockholders, or equity stakeholders	0.86	
*Organizational democracy full scale*		
Operational subscale	0.40	
Tactical subscale	0.98	
Strategic subscale	0.90	
**Meaningful work** (Steger et al., 2012)		
*Subscale: Positive meaning*		
1.I have found a meaningful career	0.78	
2.I understand how my work contributes to my life’s meaning	0.87	
3.I have a good sense of what makes my job meaningful	0.84	
4.I have discovered work that has a satisfying purpose	0.81	
5.I view my work as contributing to my personal growth	0.41	
*Subscale: Meaning making through work*		
5.I view my work as contributing to my personal growth	0.45	
6.My work helps me better understand myself	0.82	
7.My work helps me make sense of the world around me	0.82	
*Subscale: Greater good motivations*		
8.My work really makes no difference to the world. (R)	0.91	
9.I know my work makes a positive difference in the world	−0.51	
10.The work I do serves a greater purpose	0.81	
*Meaningful work full scale*		
Subscale: Meaning making through work	0.83	
Subscale: Positive meaning	0.85	
Subscale: Greater good motivations	0.88	
**CSR Perceptions** (Lee et al., 2017)		
*Subscale: Philanthropic CSR activities*	Time 1	Time 2
Our company helps solve social problems	0.76	0.71
Our company has a strong sense of corporate social responsibility	0.76	0.72
Our company gives adequate contributions to local communities	0.76	0.71
Our company allocates some of their resources to philanthropic activities	0.65	0.61
Our company plays a role in society that goes beyond the mere generation of profits	0.72	0.72
Our company encourages its employees to participate in voluntarily activities	0.71	0.70
Our company emphasizes the importance of its social responsibilities to its employees	0.84	0.82
Our company organizes ethics training programs for its employees	0.71	0.69
*Subscale: Ethical CSR activity*		
Our employees are required to provide full and accurate information to all customers	0.71	0.68
Our company has a comprehensive code of conduct	0.80	0.76
Our company is recognized as a trustworthy company	0.65	0.62
*CSR perceptions*		
Subscale: Philanthropic CSR activities	0.89	0.99
Subscale: Ethical CSR activity	0.77	0.76

We tested the full hypothetized model in a Structural Equation Model that showed satisfactory fit indices (*χ*^2^(883) = 1414.07, RMSEA = 0.053, CFI = 0.91). The results support the hypotheses (see Figure 1). First, the results confirms that the more organizational democracy that an employee experice, the more meaningful is work perceived to be (support for Hypothesis 1). Second, the higher the organizational democracy, the higher CSR perceptions (Hypothesis 2), and, in turn the more meaningfulness at work does a person experience (Hypothesis 3). Finally, the indirect effect (unstandardized) was estimated to be 0.46 and the biascorrected bootstrap 95% confidence intervals ranged from 0.17 to 1.1. The full effect was 1.36 [CI from 0.67 to 2.27]. These results confirm Hypothesis 4 about a partial mediation effect, i.e., that the relationship between Organizational Democracy and Meaningful work can in part be explained by CSR perceptions as an intermediate variable. The SEM model with controls showed that none of the control variables were significantly related with the other variables, the hypothesized relationships did not change substantially and were not insignificant in the model with control variables. Therefore, the results could not be due to third variable confounding effects of age, gender or education.

## Discussion

The aim of this study was to test a mediation model to explore the direct effect of organizational democracy on meaningful work and to disentangle the mediating effect of the employees’ CSR perceptions. The results indicated that organizational democracy has a significant direct effect on meaningful work, in line with Hypothesis 1. Regarding Hypothesis 2, our results showed that organizational democracy was related to CSR perceptions, so Hypothesis 2 was supported. Moreover, Hypothesis 3 was confirmed as CSR perceptions significantly predicted the experience of meaningful work. Lastly, our results showed that CSR perception partly mediated the relationship between organizational democracy and meaningful work, supporting Hypothesis 4.

### Theoretical contribution

This study offers three key contributions to both theory and research on organizational democracy, CSR, and meaningful work. First, we believe that our study is the first to directly examine the relationship between organizational democracy and meaningful work. Thus, the study aligns with and lends empirical support to a relationship that has historically been given theoretical attention, but lacks empirical exploration (Arneson, 1987; Yeoman, 2014). Our study is in alignment and extends the longitudinal study by Martela et al. (2021), who find that the experience of autonomy was positively related to the experience of meaningful work. Organizational democracy may be one such important source of autonomy and may thus be considered an important contextual source of autonomy. Our study extends the work by Martela et al. (2021) further, by investigating how contextually experienced autonomy trough organizational democracy may affect the experience of meaningfulness in organizations. The present study also highlights the relative importance of organizational democracy in general, illustrating how organizational democracy may be important for stimulating individual-level effects. This is in accordance with the meta-analysis by Weber et al. (2020), who found that organizational democracy significantly predicts beneficial individual level outcomes. Our study adds to this finding and demonstrates how the perception of meaningful work may be another positive and notable individual-level effect of organizational democracy.

Second, our study underscores the importance of organizational democracy in shaping the employees’ view of the organization. Previous research has found that CSR perceptions are related to meaningful work (Chaudhary, 2020; Youn and Kim, 2022). Our study aligns well with those findings by demonstrating a positive link between CSR perceptions and the experience of meaningfulness. However, we also extend these findings as the present study is the first to investigate and find a positive relationship between organizational democracy and CSR perceptions. This finding aligns and extends the findings of Jin and Drozdenko (2010) who find that power sharing, information sharing, and democratic ideology is related to CSR in organizations. Our results complement this finding by showing that organizational democracy, which entails power sharing, knowledge sharing, and democratic ideology is indeed related to CSR in organizations. However, our findings also extend these results by showing that democratic organizational contexts also work at an individual level by increasing the employees perception of philanthropic and ethical CSR activities. Moreover, to the best of our knowledge, our study is the first to apply CSR as a mediating mechanism in the relationship between organizational democracy and meaningful work. This finding aligns well with the argument made by Unterrainer et al. (2011) that organizational democracy has a socializing effect on the employees, and that organizational democracy may contribute to a more favorable global view of the organization. Although the present study does not measure the actual CSR activities of the company *per se*, one could argue that actual CSR activities and CSR perceptions should be strongly related. This, in turn, may suggest that democratic organizations could be better equipped to promoting CSR activities, and thus contribute to a more socially responsible business world in general.

Third, our study also contributes to the literature on meaningful work and especially the call for research on which organizational antecedents promote the experience of meaningful work. Our results show that there is indeed a positive, significant relationship between organizational democracy and meaningful work, suggesting that organizational democracy may be an important contextual factor that contributes to the experience of meaningful work. Thus, our study responds to the research gap presented by Rosso et al. (2010), by giving more knowledge on how different sources of meaningful work simulate the experience of meaningfulness in the workplace simultaneously. Moreover, our findings are in alignment with the theoretical model proposed by Ashford and Pratt (2003), which highlights the importance of employee involvement practices in the understanding of how to create meaningful work.

Compared to other relevant research regarding contextual antecedents of meaningful work, our study is in alignment with the empirical work of Peng et al. (2016), who find that a *leader context* that grants autonomy trough intellectual stimulation, increases the perception of meaningful work from the employees. Our research expands this study by looking at how autonomy granted trough the specific *organizational context* that the leader operates within, affects the experience of meaningful work. In general, our study therefore underlines how it is not only the *work tasks* that shape the employees experience of meaningfulness, but also the *organizational context* that the employees operate within. This is in accordance with the qualitative research by Bailey and Madden (2017) who conclude that *“all jobs have the potential to be both meaningful and meaningless”* (p. 3), and that meaningfulness would arose through work experiences that were shared and autonomous. These findings align well with the result of our research as sharing and autonomy may be well stimulated through a context that fosters organizational democracy.

### Practical implications

This study has several practical implications that are worth noting. First, we show that if organizations wish to create meaningful workplaces, they should not only focus on the work tasks that the employees are given, but also focus on the organizational context that the employees operate within. The study reveals that organizational democracy is significantly positively related to the experience of meaningful work, suggesting that fostering organizational democracy in organizations can serve as an important mechanism to improve the experience of meaningful work. However, our study also suggests that organizational democracy may be a important factor to improve the perceptions of CSR in the organization, which strengthens the experience of meaningfulness. Thus, organizations that wish to improve employees’ experience of meaning and perceptions of CSR should strive to create structures where participatory decision making is mandatory, both at the operational and short-term level and at the long-term strategic level.

### Limitations and further research

Although our study has several strengths, such as an original theoretical contribution and a sample representing diverse industries, it also has certain limitations that are worth noting. First, the data in our study come from only one source − the employees − so the study may be subject to common method bias. Thus, the employees in our study might for example have rated CSR perceptions high, based on their previous high rating of organizational democracy, or because they are generally satisfied with their workplace (Podsakoff et al., 2012). However, Spector (2006) and Podsakoff et al. (2012) found that common method bias may be an overrated problem in general. Moreover, in our sample we separated the collection of the independent and the dependent variables, which is found to reduce common method bias (Podsakoff et al., 2012). A second limitation of the study is the possibility of reversed causality in our data. For example, it could be that the experience of meaningfulness also leads to greater involvement in organizational practices and therefore create a perception of a more democratic organization. Therefore, further studies should aim to explore the causality between the constructs further by using objective data or experimental methods that are better suited to establish causality. For example, further research could explore and compare democratic organizations with less democratic organizations to explore differences in perceptions of meaning and CSR perceptions.

## Conclusion

In this study we explored the effect of organizational democracy on meaningful work and how perceptions of CSR are involved as an important mechanism in this relationship. Specifically, we found that organizational democracy is an important antecedent of meaningful work and that the employees’ perception of CSR partly mediates this relationship. Our study thus has *theoretical implications* for scholars doing research on both organizational democracy, CSR and meaningful work. Our study has *managerial and practical contributions* by showing how a specific organizational context can be arranged to stimulate CSR perceptions and meaningful work. The study has certain *limitations,* as the data obtained are cross sectional and comes from one source. We encourage *further research* to explore the effect of organizational democracy on meaningful work, with more objective and longitudinal data and to consider other intermediate variables in this relationship. Ultimately, this knowledge can be an important step toward understanding how to create more meaningful work.

## Data availability statement

The raw data supporting the conclusions of this article will be made available by the authors, without undue reservation.

## Ethics statement

Ethical review and approval was not required for the study on human participants in accordance with the local legislation and institutional requirements. The patients/participants provided their written informed consent to participate in this study.

## Author contributions

MS and TJ developed the idea for the study and revised the paper in its final form. MS collected the data and wrote the majority of the paper, notably theory and discussion sections and in part the methods section. TJ conducted the analyses and wrote the results section and parts of the methods section. All authors contributed to the article and approved the submitted version.

## Conflict of interest

The authors declare that the research was conducted in the absence of any commercial or financial relationships that could be construed as a potential conflict of interest.

## Publisher’s note

All claims expressed in this article are solely those of the authors and do not necessarily represent those of their affiliated organizations, or those of the publisher, the editors and the reviewers. Any product that may be evaluated in this article, or claim that may be made by its manufacturer, is not guaranteed or endorsed by the publisher.
